# Electrophysiological Evidence of Dissociation Between Explicit Encoding and Fast Mapping of Novel Spoken Words

**DOI:** 10.3389/fpsyg.2021.571673

**Published:** 2021-03-04

**Authors:** Yury Shtyrov, Margarita Filippova, Evgeni Blagovechtchenski, Alexander Kirsanov, Elizaveta Nikiforova, Olga Shcherbakova

**Affiliations:** ^1^Center of Functionally Integrative Neuroscience (CFIN), Department of Clinical Medicine, Aarhus University, Aarhus, Denmark; ^2^Laboratory of Behavioural Neurodynamics, Saint Petersburg State University, Saint Petersburg, Russia; ^3^Department of General Psychology, Faculty of Psychology, Saint Petersburg State University, Saint Petersburg, Russia

**Keywords:** word learning, language acquisition, fast mapping, explicit encoding, event-related potential, electroencephalography

## Abstract

Existing behavioral, neuropsychological and functional neuroimaging data suggest that at least two major cognitive strategies are used for new word learning: fast mapping (FM) *via* context-dependent inference and explicit encoding (EE) *via* direct instruction. However, these distinctions remain debated at both behavioral and neurophysiological levels, not least due to confounds related to diverging experimental settings. Furthermore, the neural dynamics underpinning these two putative processes remain poorly understood. To tackle this, we designed a paradigm presenting 20 new spoken words in association with pictures in either FM or EE settings, closely matched for auditory and visual features and overall task demands. We tested word acquisition using a range of behavioral measures as well as passive event-related potential (ERP) responses, an established measure of word memory trace activation, and compared brain activity elicited by novel FM and EE words before and after the learning session. Behavioral data obtained in free recall, recognition and semantic word-picture matching tasks indicated successful acquisition of new words after just 10 exposures. Crucially, we found no behavioral evidence of different acquisition outcomes between FM and EE learning. ERP data, which exhibited the main response peaks at ~170, 250, and 520 ms, also indicated successful learning, with statistically different responses between novel and familiar words present only before, but not after the training, suggesting rapid formation of new neural memory circuits matching in activation those for previously known words. Furthermore, already at the earliest peak, we found different topographic distributions for the two learning types, with left-lateralized FM dynamics, suggestive of core language system involvement, and more diffuse activity for EE items, possibly suggesting the role of attention/executive control network. A similar effect also manifested later, at ~520 ms. Our data suggest that while both EE and FM learning can be successful for rapid word acquisition at the behavioral level, the diverging electrophysiological patterns suggest a dissociation between the neural systems underpinning these learning strategies.

## Introduction

The ability to communicate using language is a specific cognitive faculty that makes humans unique among all animal species on the planet. In spite of the fundamental role that language plays in our individual lives and social well-being, its biological origins and its cognitive and neural mechanisms are still poorly understood (Corballis, 2009). Critically, there is a dearth of knowledge of specific language acquisition mechanisms that underpin our extremely efficient ability to learn a large number of new words which humans exhibit most vividly as children at different stages of development, as well as later, as adults when learning a new language or acquiring new lexical items in the native tongue.

The mechanisms underpinning word acquisition, at both behavioral and neural levels, remain a topic of debate (for review, see e.g., Dollaghan, 1985; Davis and Gaskell, 2009). At the systems level, the process of learning is usually separated into initial encoding and later consolidation. The former has been shown to occur rapidly and involve multiple brain areas, most crucially medial temporal lobe (MTL) including hippocampus and parahippocampal cortices, whereas the latter is a more gradual process leading to the formation of long-term memory traces in the neocortex (McClelland et al., 1995; Norman and O’Reilly, 2003). This dual-stage approach, most commonly known as the “complementary learning systems” framework, is supported by a variety of investigations, from animal studies with hippocampal and cortical lesions differentially affecting the two stages (Talpos et al., 2008), to patients studies with hippocampally-damaged amnesiacs that demonstrate specific patterns of retrograde memory loss (Scoville and Milner, 1957; Sharon et al., 2011), to computational models simulating these processes using artificial neural networks (O’Reilly and McClelland, 1994). This framework has been extended to account for word learning mechanisms, based on key experiments, which suggested that newly-learnt word forms fully enter the lexicon only after an overnight consolidation period accompanied by changes in neocortical and MTL activity (Gaskell and Dumay, 2003; Davis and Gaskell, 2009; Himmer et al., 2017). While this approach can successfully explain a range of phenomena in the fields of memory, learning, and language, a body of observations also suggests the existence of an MTL-independent route for direct acquisition of new word forms by the neocortex, at least under certain conditions (see Shtyrov, 2012, for review, and below).

In real-life situations, acquisition of new words can arguably be achieved through two main learning strategies: a direct explicit instruction (for instance, “This is a *nonie*, try to remember it”) or a contextually-driven implicit inference/deduction (“There is a cup, a kettle and a *nonie* on the desk. Give me the *nonie*, please”). Explicit learning, often dubbed *explicit encoding* (*EE*), is usually associated with repetitive presentation occurring over extended (or even multiple) practice sessions, such as classroom instruction or rehearsal, and can thus also be characterized as intentional learning (Konopak et al., 1987; Shtyrov et al., 2019). In contrast, contextually-driven deduction normally takes place in routine daily interactions between individuals and appears to have a near-immediate effect, evident before long-term memory consolidation processes set in. It requires very few encounters with the new word (possibly as few as one single exposure) provided that the context promotes indirect inference through exclusion or deduction (Bloom and Markson, 1998; Halberda, 2006; Horst and Samuelson, 2008; Vasilyeva et al., 2019). Such rapid context-driven implicit acquisition, which relies on contextual inference (and can thus also be characterized as incidental learning), is known as *fast mapping* (FM; Carey and Bartlett, 1978) and appears to be a general learning mechanism that plays a key role in acquiring new words in the process of natural language learning.

Although it continues to function throughout life, FM seems to be most efficient in children at the early (pre-school) stages of life (Rohde and Tiefenthal, 2000; Bion et al., 2013). Crucially, hippocampus and episodic memory are not fully developed at this age (Bauer, 2008), indicating that FM cannot benefit from medial temporal memory systems. Indeed, FM (unlike EE) has been suggested to be less dependent on MTL and hippocampo-neocortical consolidation circuits and to be reliant mostly on the neocortex directly (Shtyrov, 2012). For instance, clinical investigations have shown that while explicit exposure (in EE fashion) of patients with MTL lesions to new information (such as novel word-picture associations) results in poor behavioral outcomes, FM learning regime leads to successful word acquisition in such patients, which, on the other hand, is hampered by neocortical damage (Sharon et al., 2011; see also Warren and Duff, 2014; Warren et al., 2016). Furthermore, FM, in contrast to EE tasks, activates a more widespread neocortical network during encoding, which seems to consistently include temporal areas, particularly anterior-temporal lobe (ATL), as shown by functional magnetic resonance imaging (fMRI) in healthy adults (Atir-Sharon et al., 2015; Merhav et al., 2015). Neocortical structures in ATL/temporal pole have, in turn, been posited as a seat of lexico-semantic representations, playing the role of a central “hub” in distributed word memory circuits (Patterson et al., 2007). Crucially, whereas EE seems to benefit from an overnight consolidation stage, FM learning does not trigger overnight changes in brain representations (Merhav et al., 2015). It stands to reason that such distinct brain signatures of the two learning strategies imply different underpinning mechanisms, explaining diverging learning dynamics and efficiency.

Despite such crucial findings delineating the main learning strategies, a number of questions and controversies remain open. For instance, findings of any advantages offered by FM and/or differential learning outcomes of the two regimes have been questioned by some studies that failed to replicate them (see Greve et al., 2014; Cooper et al., 2019). Furthermore, in spite of frequent claims of FM benefits, most of the above studies have in fact showed better recognition rates for new words learnt in EE tasks (although this *per se* does not undermine putative distinctions between the EE/FM brain mechanisms). Another important limitation is the lack of control over the overall conditions when comparing the two strategies experimentally: the behavioral routines typically used to contrast the learning regimes differ in more than one dimension. A typical paradigm in such studies (as in, e.g., Merhav et al., 2015) involves a word-picture association task, in which the FM condition (“*Does nonie have spikes?*”) presents the subject with two or more images, only one of them being novel, implicitly requiring the subject to infer that the new word refers to the unfamiliar image; the EE condition, however, usually presents only a single image in conjunction with its name (“*This is nonie*”). Such a design implies a lack of basic visual balancing between the two conditions, which puts differential load already at the level of initial visual processing of the stimuli. Furthermore, at the higher cognitive level, it creates different distribution of attention across the visual field for the two conditions. Although attention obviously plays an important role in learning, the attention mechanisms are not part of the language system as such and should be disentangled from it, and attention-related experimental confounds should be minimized. Third, while these two conditions inevitably frame the task in cognitively different manners (which is unavoidable), it is further exacerbated by the way the instruction is typically offered in such experiments. Typically, in FM condition (Carey and Bartlett, 1978; Atir-Sharon et al., 2015), a Question (“*Does nonie have spikes?*”) or a Request (“*Bring/show me the chromium tray*”) are used, while Naming is used in EE (“*This is nonie*”). Pragmatically, these three tasks constitute different *speech acts* (Searle, 1969), which put different demands on the cognitive system and are underpinned by overlapping yet different brain networks (Van Ackeren et al., 2012; Egorova et al., 2014). Clearly, this further confounds any distinctions found between FM and EE in behavioral outcomes or neurophysiological activation patterns. It may not be possible to fully balance the two regimes without removing intrinsic distinctions between them; yet, it seems highly important to minimize the effects of such confounding factors as visual features, attention, cognitive load, and contextual framing, in order to disentangle their mechanisms with more certainty. An attempt at this was made in the present study.

Importantly, the bulk of previous research addressing the FM-EE distinction was done behaviorally and/or using slow neuroimaging tools, such as fMRI. These measures do not have the necessary resolution for assessing rapid neuronal activations that are known to take place on the millisecond range; this is particularly important for the language function, which relies on temporally dynamic processing of information that rapidly unfolds over time (Friederici, 2002; Pulvermüller et al., 2009; Shtyrov and Stroganova, 2015). To better understand the neural processes underpinning different types of language learning, there is a need for a more direct measure of electric neuronal activity; this can be provided by time-resolved imaging tools such as electroencephalography (EEG), which was used as a method of choice in the current experiment.

We set out to fill these gaps in an EEG experiment we report below. To this end, we designed a naturalistic paradigm in which the participants had to learn 10 new words through either EE or FM presentation of new spoken word forms and objects in an audio-visual context. Acoustically, phonologically, and orthographically matched familiar words were used as controls. These spoken forms were paired with visually presented images of familiar or novel objects, which were fully controlled for their basic physical features. Two images were presented in all conditions, which were either familiar-unfamiliar pairs in FM, or a novel object beside a senseless image in EE (all images being matched for basic visual properties across all conditions, see Materials and Methods). To match the conditions for the pragmatic use of language, both FM and EE employed a question, while still promoting different learning strategies, for example: “This is nonie – will you recognize it later?” (EE) or “Does nonie have horns on its head?” (FM), both requiring a yes/no answer (English examples are given here for illustration only, see Materials and Methods for more details). Each word was presented 10 times in 10 different sentential contexts, paired with 10 different images of the same type of object (for instance, different images of a previously unknown type of tool). The learning outcomes were tested in a comprehensive range of behavioral tasks at lexical and semantic levels.

To address the neural activation elicited by the new words, we recorded passive auditory event-related potentials (ERPs) elicited by the novel items. Passive (i.e., not requiring subject’s overt attention or stimulus-oriented tasks) paradigms are known to be a reliable tool for assessing the status of stimuli in the long-term lexical storage: the amplitude of early (<200 ms) passive ERP responses to spoken words is enhanced in comparison with acoustically matched pseudowords, and this enhancement is believed to be a neural signature of an automatic word-specific memory trace activation underpinned by robust connections in lexical memory circuits (Pulvermüller and Shtyrov, 2006; Shtyrov et al., 2010; MacGregor et al., 2012). This approach has been repeatedly used to address the lexico-semantic stimulus properties and the dynamic processes of word memory trace build-up in the brain: an early increase in the ERP amplitude, which was found by comparing pre-learning vs. post-learning brain responses or by tracing them throughout the exposure, has been linked to the increasing familiarity, likely underpinned by the rapid build-up of a new neural memory circuit for the novel word form (Shtyrov et al., 2010; Shtyrov, 2011; Kimppa et al., 2016; Partanen et al., 2017, 2018; Bermúdez-Margaretto et al., 2020). In conjunction with the above carefully balanced experimental design enabling our participants to learn new words using two different strategies, we recorded ERPs to these items immediately before and after the short learning session, and compared the resulting ERP dynamics between the recordings and between the learning regimes.

## Materials and Methods

The experiment included four main parts: (1) passive EEG recording of auditory responses to familiar and novel word forms prior to learning, (2) learning task, (3) second EEG session immediately after the learning, and (4) behavioral assessment of the learning outcomes. All stimulus presentation was controlled in NBS Presentation v20.0 environment (Neurobehavioral Systems, Berkeley, CA).

### Experimental Participants

Twelve volunteers (10 females; 17–34 years old; mean age = 25.2, SD = 4.88) took part in the study. All participants were monolingual native Russian speakers, right-handed (handedness established using Edinburgh Handedness Inventory; Oldfield, 1971), had normal or corrected to normal vision, and no history of neurologic or psychiatric disorders or drug abuse. They gave an informed consent approved by the SPbU Ethics Committee and were remunerated for their time.

### Stimuli

The main stimulus set included 40 word forms, 20 of which were real nouns of the participants’ native language and the other 20 – novel forms, matched with the real words for a number of parameters and highly similar to them phonologically. To create the stimuli, we first selected 20 real Russian words, which (1) were frequent nouns with commonly known meanings, (2) phonologically, had tri-phonemic consonant-vowel-consonant (CVC) structure, and (3) orthographically, were written in three letters. The resulting list of words included highly recognizable items [e.g., *myach* (*мяч – ball*), *byk* (*бык – bull*), etc.]. We then composed novel triphones by recombining the onsets and offsets of these real words: for instance, real words *fen* (*фен – hairdryer*) and *mul* (*мул – mule*) were used to produce novel word forms *fel* (*фел*) and *mun* (*мун*). That implies that, on average, both familiar words and new word forms included the same phonemes combined differently, controlling for purely acoustic differences between the stimulus types. All items were grouped into lists of five for further counterbalancing across conditions (see below). These quintets were balanced for diphone frequencies (separately for the first two and the last two phonemes), and, within the word sets, for the lexical frequency and familiarity. We used Russian National Corpus psycholinguistic database^1^ to estimate stimuli’s properties.

All auditory stimuli (novel and familiar word forms, as well as contextual questions) were digitally recorded using a female native speaker of Russian. Their length and volume were balanced across conditions using Adobe Audition 1.5 software (Adobe Systems, San Jose, CA).

**Figure 1:**
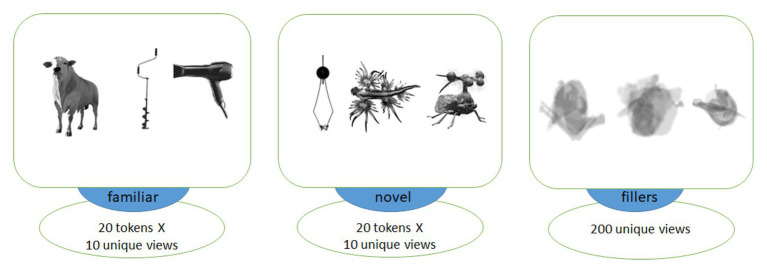
Examples of images of objects used in association with familiar **(left)** and novel **(middle)** words as well as visually matched filler items **(right)**. Each trial included two images from different categories, with filler images used in the explicit encoding (EE) condition only to balance it visually against the fast mapping (FM) one.

To compose visual stimuli corresponding to the spoken word forms, we used photos of 20 familiar and 20 novel objects. For control familiar items, images of common objects implied by our stimuli were used (e.g., bull, knife, ball, owl, etc.). As novel items to be learnt, we used images of animals or inanimate objects not known to our typical experimental participants. As inanimate novel objects, we used images of ancient or rare tools or musical instruments, whereas novel animals were, for example, deep-sea or other rare creatures (see Figure 1 for examples). Evaluation of the stimuli by independent raters prior to the experiment established that all familiar stimuli were recognizable by each rater, and that novel stimuli were not. There was an equal number of animate and inanimate objects in both stimulus subsets.

We chose 10 different unique images for each object, such that each token was presented only once to each subject in the learning block. These graphically different tokens were made from five different actual variants, with two views of each individual item, to ensure both generalizability and similarity across each set of 10 pictures, making sure the subjects learnt them as novel types of animals/objects, rather than a proper name of one specific individual item. All images were converted to monochrome, placed against white background (400 х 400 pixels) and centered. We then applied noise contamination, scaling, rotation and overlapping to compose, out of these original images, a set of blurred filler images without clearly identifiable content, which were used to balance the EE condition visually against the FM one. The average luminance for all stimulus types was balanced by calculating and matching the number of white and black pixels in each image across all stimulus types; *t*-tests revealed no significant luminance differences between any of the three groups of images (all values of *p* > 0.7).

### Learning Paradigm

**Figure 2:**
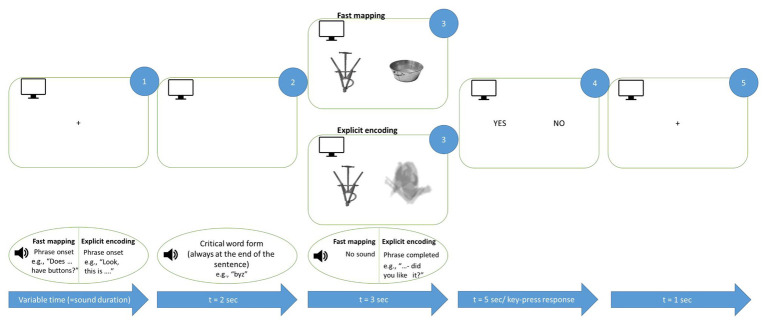
Schematic illustration of FM and EE learning trials. Auditorily presented sentences were accompanied by graphically presented objects corresponding to new words. The critical word was always the last auditory stimulus before the image onset (see Materials and Methods). Both EE and FM conditions included a question and were maximally matched for their overall properties. Note that English examples are given for illustration only, the original sentences were produced in accordance with Russian grammar.

We used an active semantic learning paradigm, which included two conditions, aimed at acquiring new words either implicitly (FM condition) or explicitly (EE condition), that were maximally matched for presentation mode but differed in terms of the participants’ exact tasks. Each condition included a picture of a target stimulus presented along with another image, and was accompanied by an auditorily presented question related to the picture (Figure 2). Under the FM condition (50% of all trials, randomly distributed), a participant had to use contextual information to infer the referent of the novel word. We used spoken questions like “Is XXX made of paper?” or “Does XXX have ears?”, whereas images of both a familiar object (for instance, a bird or a football) and an unfamiliar novel one appeared on the screen side by side. Thus, in the FM condition, the participant was to infer the target object by excluding the other (familiar) object presented simultaneously. Under the EE condition (50% of trials), the learner’s goal was to become explicitly familiarized with the target object and the corresponding word. To this end, the object was explicitly introduced, and was accompanied by a question in order to make the presentation mode similar to the FM condition, but, importantly, without the need for implicit inference, for example: “Take a look at XXX – will you remember it?” or “Here is XXX – have you had a good look?” In the EE condition, instead of a familiar competitor object side-by-side with the target one, a blurred filler image was presented, matched in overall size and luminance with the meaningful images (see Figures 1, 2), in order to make it visually balanced against the FM task. The EE and FM trials were presented in the same pseudo-random sequence, to avoid different attentional or strategic biases that might arise in case of blocked presentation.

We counterbalanced the novel word forms such that, during the experimental procedure performed with different participants, each novel triphone acted in different roles: as novel animal in EE or FM conditions, novel inanimate object in EE or FM conditions, or as an untrained pseudoword (used as foils in post-learning testing, see below). Contextual questions for both conditions were composed such that each trial had a unique combination of word form/picture/question. The number of words in these sentences was matched between EE and FM conditions.

As a control for the learning conditions, the same number of matched real words and corresponding pictures of real objects were presented in an identical fashion, also broken into the explicit and implicit presentation modes, with similar questions asked about them. Ten items for each category were used (making 40 items in total), combining the variables of familiar/novel and EE/FM learning type in a counterbalanced fashion. For variety, each category included non-living objects (e.g., a hairdryer and an unknown tool) and living animals (e.g., a fish and an unknown living creature) in equal numbers.

Each item was used 10 times in the learning block, but the particular image (specific tokens and viewing angles) and the auditory phrase varied from trial to trial. Different stimulus types and learning settings were pseudo-randomly mixed in one training block broken into three sub-blocks (to reduce fatigue), which were interleaved with passive presentation (audio only, no contextual training) of 10 matched control pseudowords that later served in the behavioral recognition tests (see below) to assess the effects of the semantic learning in comparison with the same number of meaningless auditory exposures.

Throughout the experiment, the participants were seated in a comfortable chair inside soundproof electrically shielded room (Neuroiconica, St. Petersburg, Russia). They were instructed to relax, refrain from unnecessary movements, listen carefully to the auditory stimuli and focus their visual attention on the computer screen in front of them. Each trial (Figure 2) started with a fixation cross on a computer screen accompanied by a spoken question in the FM condition (for instance, “Does XXX have pointy ears?”) or by a direct naming in the EE trials (e.g., “This is XXX in front of you.” Note that English examples are given for illustration only. The original Russian sentences were matched in length across conditions; the critical word was always the last one in the sentence and in nominative case). Then, two images, one of them being the target, were displayed for 3 s. In the FM condition, images of novel and familiar objects were presented side-by-side such that the subject had to infer which of the two the question referred to. In the EE condition, one of the images was a blurred filler stimulus such that the introduced object could be identified unambiguously; the blurred filler was used to make the two conditions visually similar and was never repeated to prevent its learning. The EE condition also included a question (such as “have you had a good look?” or “did you like it?” – note that all questions in both conditions were unique, without repetitions), which appeared during the image presentation but, unlike FM, did not imply any feature inference. The left/right presentation of the target stimulus was balanced across all stimulus types; each trial was graphically unique. Finally, the screen with response options (yes/no) appeared for both conditions; the response was given by the left index finger using a response pad (RB-740, Cedrus, San Pedro, CA).

### Passive Listening and EEG Recording

To evaluate learning-related changes in the brain’s responses to novel items, we recorded EEG during a passive listening task, which was performed before and after the semantic training. Participants were instructed to focus on watching a silent cartoon presented on a computer screen without any subtitles or other texts,^2^ while the auditory stimuli were presented *via* stereo headphones. All familiar and novel word forms were repeated 10 times each in a pseudorandom sequence (among the same number of similar filler items), making up 100 trials per each category, with stimulus onset asynchrony (SOA) jittered between 1100 and 1200 ms. EEG was recorded using ActiChamp 128-channel active EEG system (BrainProducts, Gilching, Germany) and PyCorder recording software (Brain Products) with 0.016–1000 Hz frequency band and 5 kHz sampling rate, with FP1 as a reference channel. The electrodes were placed in a cap according to the extended 10–20 system (M1 montage; EasyCap, Herrsching, Germany).

### Behavioral Assessment

After the training procedures and EEG recording, we assessed the quality of novel word acquisition using three different tasks. These included free recall, auditory recognition, and word-picture matching tasks.

In the free recall task, the participant had to list as many word forms presented during the experiment as possible. The number of words to recall and the time were not limited; however, most participants completed this task in about 5 min. The participants wrote down the words, and we counted the number of correct namings; this quantification was straightforward given that Russian is transparent with respect to phonology-orthography correspondence (particularly for monosyllabic words with a single stressed vowel, as those used here).

In the auditory recognition test, the participants’ task was to identify whether they had encountered the stimuli earlier during the experimental session. After the auditory presentation of each stimulus, the participant pressed “yes” button with their left index finger in case they believed that the stimulus had been presented earlier, and “no” button in case they did not. This block included all the stimuli that appeared earlier during the experiment, and an equal number of two types of acoustically and phonologically similar foils: (a) words (also matched semantically: tools or animals) and (b) untrained pseudowords, presented pseudorandomly. The button press was followed by a pause of 500 ms and, then, by the next stimulus presentation. Response timeout was set to 5 s.

The semantic word-picture matching task was implemented as a forced choice between four alternatives. Here, the target auditory stimulus was presented simultaneously with four pictures presented earlier in the learning block (in a 2 × 2 format), only one of which corresponded semantically to the spoken sound. The task was to select the correct picture by pressing one of coded response keys using the left index finger. Each item was only presented once as the correct choice but could be used as a foil in other trials; targets and foils were balanced for semantic category across trials.

The tests were presented in the order aimed at minimizing any additional carry-over effects on learning they might prompt: free recall (not involving the actual stimuli and thus not prompting extra learning), followed by recognition (with just the word forms randomly presented among foils, without semantic references) and finally the semantic test. Importantly, even if extra reinforcement could take place through stimulus repetition, this would have been the same for both EE and FM items. Equally importantly, the EEG recording was run before and after learning, but before the behavioral assessment, so the EEG data were not affected by the assessment tests; in turn, even if the behavioral tasks could benefit from extra stimulus repetition in the EEG, this would have been the same for EE and FM stimuli, as these were played back similarly in a design that does not require (overt) attention, and thus could not bias the results in either direction.

Behavioral data were analyzed for reaction times and the number of correct responses using ANOVA and *t*-test comparisons. Particular attention was given to comparison between behavioral outcomes of explicit and implicit learning.

### EEG Analysis

All EEG/ERP analyses were performed using custom-built scripts in Matlab 12.0 programming environment (MathWorks, Natick, MA) and Berlin Brain-Computer Interface (BBCI) toolbox.^3^ First, continuous EEG signals were band-pass filtered between 1 and 45 Hz (fourth-order Butterworth filter), downsampled to 250 Hz sampling rate and re-referenced to common average reference. Visual inspection and power spectrum analysis were performed to identify and disable noisy channels (on average two channels per subject were disabled, out of 128). Ocular artifacts were removed with Fast Independent Component Analysis algorithm implemented as FastICA toolbox in Matlab (Hyvarinen, 1999). EEG recordings were segmented using stimulus event markers into epochs from −200 to 1000 ms after stimulus onset. Pre-stimulus baseline was taken at −200–0 ms before the stimulus onset. Additional cleaning of ERP segments was made based on standard deviation analyses of each trial implemented in Berlin BCI toolbox in Matlab. One participant’s dataset was corrupt due to EEG equipment failure, and all further analyses were run on remaining data.

As mentioned above, the stimuli were rotated such that the same items were paired with different EE or FM conditions and pictures. This enabled us to look at the ERPs largely unconfounded by the purely physical features of the stimuli. Passive auditory ERPs are known to reflect memory trace activation for existing words, typically seen as an enhanced negative response at latencies below 200 ms, and have been used to trace the formation of new memory traces during word acquisition, which becomes manifest as an amplitude increase of these early responses following learning (Kimppa et al., 2015, 2019; Berthelsen et al., 2018; Vasilyeva et al., 2019). Hence, these early ERPs were *a priori* of highest interest here.

For an unbiased data-driven analysis, overall activation strength of the ERPs was first quantified as the global root mean square (gRMS) of the ERP responses across all scalp electrodes, stimuli, and conditions. To this end, the grand average response was first calculated across all conditions and stimuli collapsed. Then, for each time point, the square root was calculated on the mean of squared amplitudes across all electrodes, producing a single gRMS response. Finally, the most prominent peaks in this global RMS were identified. Although this approach may be less sensitive to transient differences between conditions, it is optimal for approaching data in an unbiased way by focusing on the periods of largest neuronal activity overall and thus avoiding double-dipping in dataset comparisons. The expected early responses became most prominent at 150–190 ms, similar to previous research. In addition, the most clearly expressed peaks in the gRMS waveform were at 230–270 and 500–540 ms. The responses were overall most expressed at fronto-central electrode sites, which is usually the case for auditory ERPs. Therefore, we extracted data from an array of 45 electrodes covering this and adjacent areas (F, FC, C, CP, and P lines with nine electrodes in each on the 10/20 layout). For each of these intervals, window-average ERP amplitudes were submitted to five-way ANOVA with factors Novelty (two levels: familiar/novel) × Learning Type (two levels: EE/FM) × Block (two levels: before/after training) × Fronto-Posterior (five levels: electrode lines from F to P) × Laterality (nine levels: electrodes from left to right). Significant interactions were followed up by *post-hoc* tests.

Finally, as the analysis indicated a number of learning-related effects showing a change in response amplitudes after training, we also computed an overall contrast between responses to novel word forms presented after vs. before training and estimated neural generators of this ERP dynamics using a distributed source reconstruction algorithm. To this end, an eLORETA solution (Pascual-Marqui et al., 2011) was calculated on a realistic head shape using a boundary-element model based on the Montreal Neurological Institute MRI template in order to account for current spread through the head tissues. Source locations were constrained to the gray matter surface and the reconstruction was applied to the grand-average data to benefit from increased signal-to-noise ratio; this was done using sLORETA/eLORETA software package^4^ on the mean pre-post difference ERP at the peak response intervals indicated by the signal-space ERP analysis above.

## Results

### Behavioral Results

All descriptive results below are reported as mean ± standard error (SE). The accuracy of *recognition* for all types of trained stimuli – familiar EE (97 ± 1.5%), novel EE (81 ± 5.34%), familiar FM (94 ± 2.42%), and novel FM (67 ± 6.5%) – differed significantly (all values of *p* < 0.01) from the control set of untrained filler stimuli, which were repeated the same number of times without semantic reference (16 ± 3.63%). Furthermore, ANOVA revealed a main effect of the Novelty factor on response accuracy: significantly more accurate recognition of familiar (95 ± 1.43%) than novel (74 ± 4.3%) items [F(1,84) = 23.4, *p* < 0.001, R^2^ = 0.218]. Similarly, there was a main effect of the Novelty factor on response times: significantly faster responses [F(1,84) = 7.67, *p* = 0.007, R^2^ = 0.08] to previously familiar (1,143 ± 250 ms) than to novel (1,382 ± 506 ms) items. Importantly, however, ANOVA showed no Novelty × Learning Type interaction and no differences (all values of *p* > 0.05) in either RT or accuracy between the EE (accuracy 89 ± 3.01%; RT 1,259 ± 55 ms) and FM (accuracy 80 ± 3.98%; RT 1,265 ± 70 ms) conditions.

In the semantic *word-picture matching* task the number of correct matches for all 4 types of trained stimuli – familiar EE (98 ± 1.15%), novel EE (79 ± 5.6%), familiar FM (97 ± 1.38%), and novel FM (73 ± 5.3%) – significantly exceeded the chance level (all values of *p* < 0.01). Further analysis using a 2 × 2 ANOVA with two factors (Novelty and Learning type) confirmed accuracy [F(1,92) = 31.1, *p* < 0.001, R^2^ = 0.253] and RT [F(1,92) = 65.7, *p* < 0.001, R^2^ = 0.417] advantage for familiar items (RT 1,777 ± 50 ms; accuracy 98%, SE = 0.9%) in comparison to novel ones (RT 3,317 ± 183 ms; accuracy 76 ± 3.8%). It showed no interaction and no differences (all values of *p* > 0.3) between the EE (accuracy 88 ± 3.16%; RT 2,455 ± 180 ms) and FM (accuracy 85 ± 3.19%; RT 2,638 ± 169 ms) conditions for this task.

As expected, the overall performance in the *free recall task* was rather low, with participants recalling less than half of all items in the absence of any cues. It was nevertheless normally distributed (difference from normal distribution according to Shapiro-Wilk test n.s., W = 0.974, *p* = 0.946) and was thus analyzed further. We found a clear effect of semantic training, with the number of correctly recalled words of all four trained types of stimuli – familiar EE (2.4 ± 0.27), novel EE (1.4 ± 0.32), familiar FM (2.5 ± 0.34), and novel FM (1.12 ± 0.23) – being significantly above that for control pseudowords presented passively without semantic training (0.28 ± 0.11; all values of *p* < 0.003). A 2 × 2 factorial ANOVA (Novelty × Learning type) on the number of correctly recalled trained word forms indicated a significant main effect of Novelty [F(1,88) = 16.8, *p* < 0.001, R^2^ = 0.16], with previously familiar items being recalled more frequently than novel ones. Differences in the number of hits between EE (1.94 ± 0.22) and FM (1.81 ± 0.23) conditions were again not significant (*p* > 0.67).

### EEG Results

**Figure 3:**
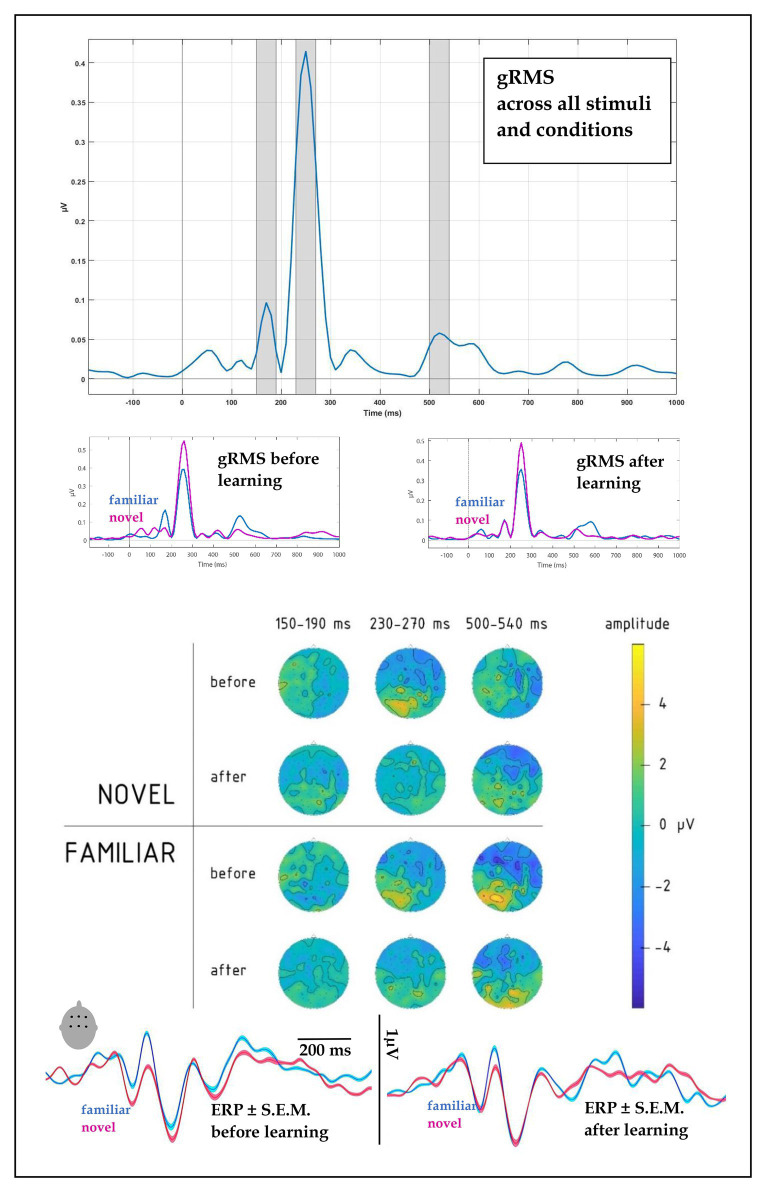
(**top**) Global root mean square (gRMS) response, computed over all conditions, subjects and electrodes, indicated the most prominent peaks at 170, 250, and 520 ms (with intervals +/−20 ms highlighted, top panel). (**upper middle**) gRMS for novel and familiar words before and after learning indicated changes in relative dynamics, particularly at the first and last peaks. (**lower middle**) Topographies of average electrophysiological responses in the 150–190, 230–270, and 500–540 ms intervals for novel vs. familiar words before and after learning session, collapsed across learning types, indicated amplitude and distribution changes after the learning block. (**bottom**) ERPs for familiar and novel words before and after learning, computed over fronto-central leads (F1, Fz, F2, C1, Cz, and C2): amplitude and SE of mean.

All stimuli elicited pronounced auditory event-related potentials in the passive listening blocks run before and after the learning session. To objectively quantify the overall response pattern, we computed an average ERP across all stimuli, conditions, and volunteers, and subjected this to RMS transformation thus reducing the entire dataset to a single timecourse (Figure 3, top panel). This global response (gRMS) showed three most prominent peaks with maxima at ~170, 250, and 520 ms. Having thus obtained unbiased estimation of the timing of overall neural activity, we subjected amplitude data from 40 ms wide windows around these peaks to statistical analysis, the results of which are reported below separately for each peak.

#### 150–190 ms Time Window

At the first peak, we found a significant interaction between Block, Novelty, and Fronto-Posterior factors [F(4,40) = 3.79, *p* = 0.011; R^2^ = 0.27; see also scalp topographies in Figure 3]. This was driven by significantly differently distributed ERPs elicited by novel and familiar word forms before learning [significant interaction of novelty and topography: F(4,40) = 6.36, *p* = 0.001, R^2^ = 0.39], whereas after the learning, ERPs for the two stimulus types no longer differed, both showing similar fronto-central negativity.

Disentangling this latter interaction further, we found that it was due to near-significant differences at fronto-central locations between the familiar and novel stimuli before training, showing larger amplitude for the familiar items [F(1,10) = 4.2, *p* = 0.068, R^2^ = 0.296], whereas after the training the amplitude for the novel items increased and this difference was obliterated [F(1,10) = 0.60, *p* = 0.811].

**Figure 4:**
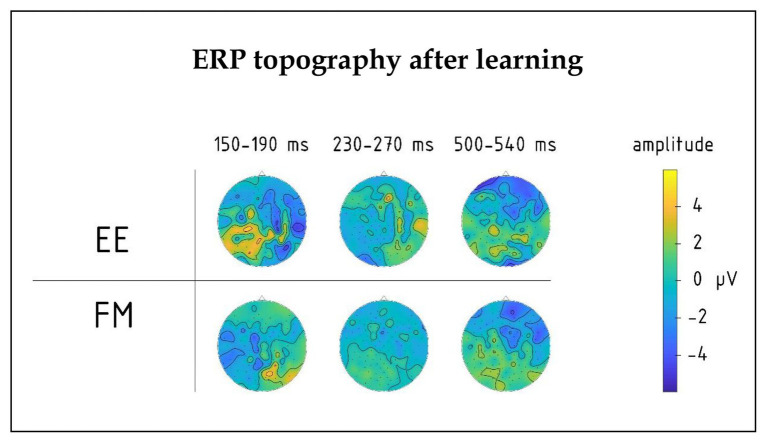
Topography of group average ERPs to newly learnt words in the 150–190, 230–270, and 500–540 ms intervals for FM and EE conditions after learning. Significant interactions involving learning type (FM vs. EE) were found in the first and third intervals, but not the second one.

**Figure 5:**
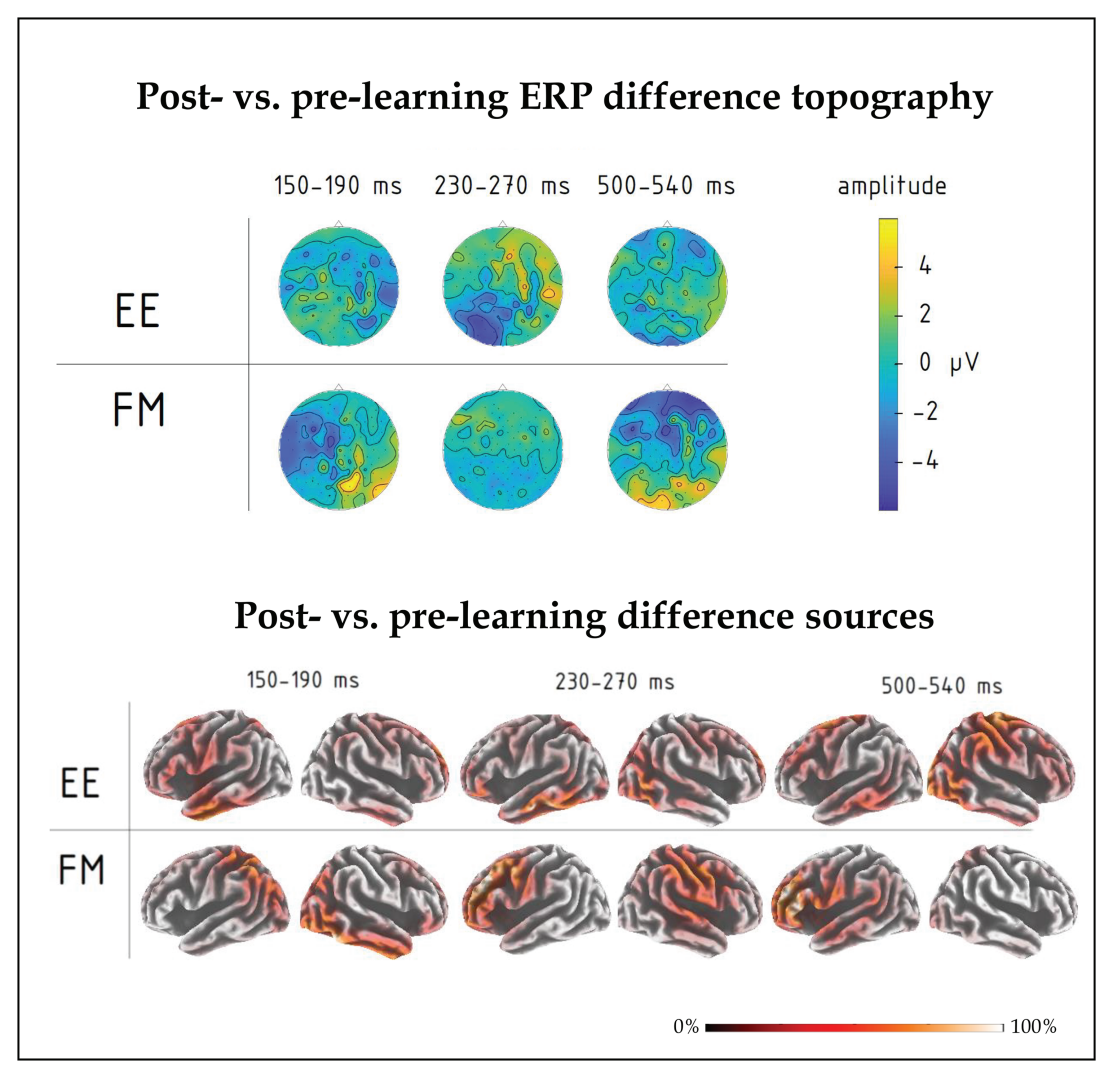
(**top**) Post-learning vs. pre-learning response difference topography of group-average ERPs in the 150–190, 230–270, and 500–540 ms intervals for FM and EE conditions. While all three time windows indicated overall learning effects, significant interactions involving learning type were found in the first and third intervals. (**bottom**) Distributed source solutions (eLORETA) for group-average post‐ vs. pre-learning difference ERPs for FM and EE conditions at the three main peaks, left-hemispheric and right-hemispheric views.

There was also a significant four-way interaction between Novelty, Learning type, Block, and Laterality factors [F(8,80) = 2.18, *p* = 0.038, R^2^ = 0.18], which was due to different pre-learning vs. post-learning lateralization dynamics of ERPs elicited by the word forms presented under fast mapping and explicit encoding conditions. Breaking down this interaction further into the data obtained before and after learning, we found that in the pre-learning recording, the interaction between Novelty, Learning type, and Laterality factors did not approach significance [F(8,80) = 0.806; *p* = 0.60]. However, after the training it became significant [F(8,80) = 2.76; *p* = 0.01, R^2^ = 0.22]: As illustrated in Figures 4, 5, for novel word forms presented under FM conditions we observed after learning a left-lateralized negativity, as opposed to more diffuse distribution for novel word forms presented under EE conditions. Breaking this interaction further down, however, did not produce significant *post-hoc* comparisons.

#### 230–270 ms Time Window

A four-way interaction between Novelty, Block, Fronto-Posterior, and Laterality factors [F(32,320) = 1.96, *p* = 0.002, R^2^ = 0.164] was observed for the time window of 230–270 ms. Breaking this down, we found that before learning, an interaction between Novelty and both topographical factors [F(32,320) = 1.98, *p* = 0.002, R^2^ = 0.165] showed that negativity of ERPs elicited by novel word forms was greater in fronto-central sites, compared to ERPs elicited by familiar word forms (see Figure 3); however, after learning, these differences between ERPs to novel and familiar word forms were no longer evident [F(32,320) = 0.82, *p* = 0.75]. No main effects or interactions involving Learning Type were found for this time interval.

#### 500–540 ms Time Window

Similarly to the preceding time window, the third peak showed an interaction between Novelty, Block and both topographical factors [F(32,320) = 1.5, *p* = 0.036, R^2^ = 0.133], which we followed up further. Before learning, the interaction between Novelty and Laterality [F(8,80) = 4.14, *p* < 0.001, R^2^ = 0.293] was driven by significantly greater negativity in ERPs elicited by familiar word forms over left frontal leads [F(1,10) = 7.1, *p* = 0.024, R^2^ = 0.414]. After learning, neither the main novelty effect [F(1,10) = 1.7, *p* = 0.223], nor its interaction with laterality [F(8,80) = 0.67, *p* = 0.72] were significant, indicating similar activity for previously known and newly learnt words.

Moreover, we found a significant interaction effect between Novelty, Learning type, and Fronto-Posterior factors [F(4,40) = 7.32, *p* < 0.001, R^2^ = 0.452] for this time window. This interaction was due to differences in topographic distributions of ERPs elicited by novel and familiar word forms under different learning conditions: in ERPs elicited by familiar words, there were no differences between FM and EE conditions [F(4,40) = 0.62, *p* = 0.65], while ERPs elicited by novel word forms indicated differential distribution between FM and EE conditions [expressed as significant interaction between learning type and topography: F(4,40) = 6.41, *p* < 0.001, R^2^ = 0.391], with a more diffuse response change for EE and fronto-central negativity increase for FM stimuli (Figure 5).

#### Source Analysis

Finally, we computed an overall contrast between responses to novel word forms presented after and before training and estimated neural generators of these ERP differences using an eLORETA distributed source reconstruction algorithm; this was done on the mean post-pre difference ERP at the peak response intervals indicated by the signal-space ERP analysis above. The results (see Figure 5) suggested the involvement of bilateral fronto-temporo-parietal networks, but indicated different patterns for FM and EE, most notably with left parietal involvement in the FM already in the first peak.

## Discussion

The analysis of behavioral data showed that one overall finding observed for all assessment tasks was the difference between previously familiar words and those trained during the experiment. Familiar nouns denoting names of well-known objects and animals showed notable performance advantages, manifest as higher accuracy and shorter reaction time in comparison to the novel words, in most of the measures applied. This is a predictable outcome since names of familiar animals and objects are regularly encountered by participants in their everyday life, whereas novel animals and objects had to be acquired and matched with their novel names in the course of the experiment. The advantage of well-known frequently used words in such measures (including different types of behavioral recognition tasks) has been well documented in previous research (Connine et al., 1990; Grainger, 1990), which corroborates the current results.

Aside from this general performance difference, the novel items were nevertheless learned successfully, as evident from the various post-learning test results obtained. When comparing their cued recognition, we found significant recognition effects (in comparison with foils) for the trained novel words, so much so that the recognition was nearly on par with that for the real words, without significant differences in accuracy (albeit still with a longer RT). Similarly, they were better recognized than non-semantisized control word forms in the free recall test. In the semantic word-picture matching task, even though the known words again showed an overall advantage, all novel items were much higher (>70%) than chance level (25%), thereby showing good semantic performance and confirming overall successful learning.

These assessments clearly indicated successful learning of the 20 new items the participants encountered in the context of 10 repetitions each, which was evident immediately after the learning block, before longer-term consolidation processes could take place. Previous studies with similarly large sets of spoken word forms (Davis and Gaskell, 2009; Dumay and Gaskell, 2012; Pulvermüller et al., 2012) indicated successful learning outcomes only after an overnight consolidation stage, even after many more (e.g., over 30) exposures. Unlike our study, which included semantic training and an array of both lexical and semantic tasks, this previous research focused on meaningless word form acquisition tested with a recognition task. The recognition task, as used in the present study (along with other tasks), also shows successful learning, but now with much fewer repetitions in the learning block. Together, these previous and present results suggest the importance of a clear semantic reference for efficient word acquisition, in line with previous research using semantically-driven training (Mestres-Missé et al., 2007, 2008; Vasilyeva et al., 2019).

Most interestingly, there were no significant differences in behavioral outcomes between EE and FM conditions. Both led to similar successful learning, without significant differences in any of our tasks, in some contrast to previous claims of differential efficiency of these learning strategies (see Introduction). This finding could be explained by the specific features of our stimulus set-up and paradigm. Unlike many previous studies, the conditions here were matched for their basic physical (auditory and visual) features. They were also matched for the overall presentation layout and resulting cognitive load, which was achieved by always requiring the participant to focus on one of the two images and to answer a question afterwards, thus requiring attention in each case as well as having a pragmatically similar situation (question answering). This balanced paradigm shows that both learning conditions lead to successful performance, which is apparent immediately after testing, even without the overnight consolidation stage. Notably, there was no disadvantage for the explicit condition, similar to another study that attempted to scrutinize the EE/FM contrast (Greve et al., 2014). This suggests that, all other factors being accounted for, both learning routes may be similarly successful.

Notably, typical developmental studies focused on FM employ a very low number of exposures with only a handful of new items. Here, we had to compromise between such traditional behavioral setups and the needs of an ERP experiment, namely, the necessity to have a large number of trials per condition in order to obtain a quality ERP signal, while at the same time having a variety of tokens to prevent ERP habituation. That led to 10 EE and 10 FM tokens to be learnt, which is on the high end of an adult person’s ability to acquire new words in a single session. Since this sizeable amount of novel vocabulary risked compromising the efficiency of learning, we increased the number of exposures to 10 per token, which helped ensure sufficient learning of these two sets of 10 tokens. In essence, while this approach still implemented a classical fast-mapping strategy, it included some modifications aimed at improving the EEG quality and balancing EE and FM conditions. Future studies could modify this approach to make the design more similar to previous FM studies using much fewer tokens and exposures (see Vasilyeva et al., 2019, for an example of a single-shot learning EEG study), particularly when applying it to developmental populations.

On the background of the successful behavioral performance in immediate measures of learning outcomes, of particular interest are the ERP results of this investigation. We compared the brain activity to novel words elicited before and after learning to address the neural changes that accompany semantic acquisition of novel items, potentially underpinning the learning process. The analysis indicated three main peaks starting already before 200 ms and taking place at ~170, 250, and 520 ms, which showed the pre-training vs. post-training modulation effects to different degrees. First, we found differences between the novel and familiar stimuli before the training block. These were expressed as larger amplitudes for familiar than for novel words before learning at both the first and the last peaks, and different topographical distributions across all three peaks, likely indicating differences in activation for novel items and pre-existing memory traces for familiar words. These differences, both in amplitude and in topography were no longer significant after the training in any of the tests, effectively equating activity for familiar items and the newly acquired ones. Along with the behavioral data, this outcome indicates successful build-up of novel memory traces for new words after just 10 encounters with them in our word-picture learning tasks, even though the number of new items (20) was substantial. This goes well in line with previous EEG and fMRI results (Mestres-Missé et al., 2007, 2008) and suggests near-immediate formation of neural memory circuits for new words in native language to be particularly efficient when a semantic reference is made available for the newly acquired words.

Such an overall response change, which putatively reflects acquisition/increased familiarity with the novel words, has been argued to be underpinned by the rapid build-up of new memory traces along the repetitive exposure (e.g., Shtyrov et al., 2010; Kimppa et al., 2015; Partanen et al., 2017; Bermúdez-Margaretto et al., 2020). Crucially, the amount of exposure and thus the resulting familiarity was the same for the two types of learning trials. In spite of this, the ERP data indicated different trajectories of these neural changes for the EE and FM conditions. These were expressed already early on in the response dynamics. Already at the first peak, we registered differential shifts in the laterality of responses to novel EE and FM items, taking place after learning. This was most visible as a negative-going dynamic over the left temporal lobe for the fast-mapping words. This is in agreement with some of the previous research, which suggested left temporal neocortex as the main hub for lexico-semantic memory traces in general (Patterson et al., 2007) and specifically for the FM of novel words into the lexicon, when acquiring them implicitly through context (Atir-Sharon et al., 2015; Merhav et al., 2015). Whereas the EEG methodology used here does not have the resolution for confirming the location of these effects with neuroanatomical precision, it highlights the time course of this activation, as taking place early on in the course of word recognition (<200 ms), at the stages that are widely considered to reflect rapid automatic activation of word memory traces (MacGregor et al., 2012; Pulvermüller et al., 2012; Partanen et al., 2017), indicating their immediate and robust formation after only a handful of exposures in a contextual semantic setting. Future studies could use more anatomically precise neurophysiological techniques (such as MR-based MEG source reconstruction) to scrutinize the structural underpinnings of the response dynamics reported here. On a more cautious side, the differences were predominantly expressed as interactions, and should be confirmed in future studies using other stimuli/languages and larger sample sizes.

The response at 250 ms did not show particular specificity with respect to the learning type; indeed, previous studies have found responses at this time to be mostly related to other types of cognitive processing (most typically, P3a, linked to attention; see, e.g., Escera et al., 1998). However, the differences in activation of memory circuits built in FM and EE settings, which became apparent in the first peak, also manifested later, in the last peak which took place at ~520 ms after the word onset. This was visible as a more broadly distributed largely negative-going response shift for explicit learning condition, while a frontal negativity, more commonly found for auditory word presentation, was registered for the FM condition. Such later responses, overlapping with the present time frame (most notably N400 and P600) are believed to reflect secondary, top-down controlled processing of linguistic input (Friederici, 2002). This may be related to the more explicit attention-controlled nature of the EE encoding, and the neuroanatomical distribution of these effects may be indicative of the involvement of the distributed fronto-temporo-parietal attention/executive system in the formation of the new memory traces under explicit instruction conditions. Previous work did link response modulation in this time frame to memory, learning, and word acquisition (see, e.g., McLaughlin et al., 2004; Bermúdez-Margaretto et al., 2020; Gosselke Berthelsen et al., 2020); this fits well with the current result, which also suggests their sensitivity to the learning mode. Importantly, while we focused on the main gRMS peaks for an unbiased data-drive analysis, future studies may also scrutinize the response timecourse for other, perhaps more transient and minute effects outside the main peaks as well as investigate links between these ERP indices and behavioral outcomes using correlation/regression analysis (Kimppa et al., 2015, 2016).

The present data may help resolve the disagreement in the literature regarding the nature of FM and its efficiency (Shtyrov et al., 2019). On the one hand, the present behavioral results cannot confirm a specific advantage of FM over the explicit learning condition, and thus to a degree corroborate similar findings of those researchers who questioned the FM efficiency phenomena (Greve et al., 2014; Cooper et al., 2019). On the other hand, our data corroborate the findings of previous clinical and fMRI studies that indicated that at least partially different brain networks contribute to word learning under these two regimes (Sharon et al., 2011; Atir-Sharon et al., 2015; Merhav et al., 2015). Crucially, in spite of this divergence in the two cerebral mechanisms, both are able to lead to successful learning to a similar degree, which explains the lack of behavioral advantage effects in some of the above studies, but still argues for the existence of (partially) different underlying brain networks.

The main advantage of our study over previous attempts in delineating the two types of learning is a very tight control over the learning conditions implemented in EE and FM tasks, ensuring to the maximum degree possible that the outcomes are not confounded by any differences between the two tasks in basic physical properties, cognitive load, visual attention, or speech act features. That said, the findings should still be taken with some caution, given some features of the experimental design and results. For instance, some studies (Berlyne and Borsa, 1968; Jepma et al., 2012) demonstrated that viewing blurred images could increase arousal, which, in turn, might facilitate learning. On the other hand, such images could also distract the participants’ attention, prompting them to resolve uncertainty in the unclear visual input (Gottlieb et al., 2013; Wade and Kidd, 2019), which could negatively impact learning. While our behavioral results do not unequivocally support either of these possibilities, it is obvious that full visual balancing of EE and FM conditions is by definition impossible, and it is hard to find a better way to control visual properties of the stimuli than the one here. Importantly, attention to both images on the screen must be at play both in EE and FM conditions (we gave the same instruction to pay attention to the screen for all conditions/stimuli), but their exact content promotes different types of learning: feature-based inference for FM and direct instruction in EE. Future studies could include additional conditions or methods (such as eye-tracking) to control for attention effects.

A similar issue relates to the use of questions, which had to differ between the two types of trials, promoting different behaviors (memorizing in EE and visual scrutiny of objects in FM). Again, these are inevitable features of EE and FM learning routes, and full balancing of the two conditions is not possible without making them the same. The present design has improved on previous studies by incorporating both conditions within the same speech act; furthermore, all questions in both conditions were unique, without repetitions, providing a variable and ecologically valid context. Further experiments are needed to disentangle effects of these specific pragmatic features of the task from those of learning as such.

The use of anatomically imprecise method here (EEG) does not allow for clear delineation of the brain structures involved. Our source analysis results suggested the involvement of bilateral fronto-temporo-parietal networks in the activity for both types of novel words. Still, they indicated different patterns for FM and EE, most notably with left parietal involvement in the FM and frontal activity for EE already in the first peak, which further confirms overlapping yet different brain mechanisms underpinning these two learning types. Source locations were calculated using grand-average data to benefit from increased signal-to-noise ratio, which such algorithms are highly sеnsitive to, particularly in the absence of individual MRIs; since this precludes statistical analysis of source data, these results should be treated with extreme caution. To address the issue of neuroanatomical substrates with more precision, future studies could use more advanced methods, such as MEG in combination with individual MRI-based source reconstruction, to resolve the putative differences between the FM and EE learning routes. That said, the use of such methods as EEG or fMRI cannot establish causal relationships between the brain structures and their function and thus only indicates indirect links between neural activity and behavior. Future studies could therefore also use non-invasive neurostimulation approaches, such as TMS or tDCS, to test the causal nature of involvement of specific brain areas in particular word learning mechanisms (Vukovic and Shtyrov, 2019; Kurmakaeva et al., 2021).

Also, whereas our present design is focused on comparing neural memory trace activations before and after learning, future research could also use similar methods to scrutinize brain responses during the learning itself (as, e.g., in Shtyrov et al., 2010); this, however, would require more trials and/or tokens to ensure sufficient signal quality, which might interfere with the task. We tried such an analysis here *ad hoc* by looking at the ERPs in the beginning and end of the learning block. Whereas this showed a tendency for an interaction between exposure time and learning type, which again suggests diverging brain mechanisms for these learning regimes, this was not significant, likely due to reduced SNR for these responses, which were based on a relatively small number of trials. This dynamic therefore remains to be investigated in future studies in more depth.

The limited sample size used in this study, while in line with many neurophysiological investigations, means that, even though the reported effect sizes indicated sufficiently robust effects, the present outcomes (at least the behavioral ones) must be treated with caution. Future studies should use similarly balanced learning conditions with larger experimental samples, as well as different stimuli and languages, to validate our findings. Finally, with our main goal being to test immediate learning effects, the experiment was not set up to assess possible differences in longer-term retention and consolidation of FM and EE items electrophysiologically; this could be addressed in the future by testing participants at different delays (overnight/days/weeks) after learning.

To conclude, we tested the mechanisms of word acquisition under two major learning modes – EE and FM – using a carefully balanced paradigm and a range of behavioral measures and ERP responses. The results indicate that while both learning modes can be successful for rapid word acquisition, their respective electrophysiological patterns diverge, which suggests a dissociation between the neural systems underpinning these learning strategies.

## Data Availability Statement

The datasets presented in this article are not readily available and may be provided on request, provided the local research ethics and data protection rules and legislation are adhered to and allow for this. Requests to access the datasets should be directed to YS, yury@cfin.au.dk.

## Ethics Statement

The studies involving human participants were reviewed and approved by St. Petersburg University Ethics Committee. The patients/participants provided their written informed consent to participate in this study.

## Author Contributions

YS, OS, and MF conceived the research. AK, EN, OS, MF, and EB carried out the research. MF and EB analyzed data. YS and MF wrote the manuscript. All authors reviewed the paper. All authors contributed to the article and approved the submitted version.

### Conflict of Interest

The authors declare that the research was conducted in the absence of any commercial or financial relationships that could be construed as a potential conflict of interest.

## Footnotes

^1^http://www.ruscorpora.ru/

^2^These were different episodes of the same silent series (*Mole [O krtkovi]* by Zdeňek, https://www.imdb.com/title/tt0841927/), randomized across the group. This made sure they were visually highly similar, but had different content to prevent familiarity and memorization of the specific episode (a confound that would arise in case of its repetition). In line with the research using similar passive paradigms, the visual distractor task was not stimulus locked, minimizing its effect on any differences between ERPs to different stimulus types.

^3^https://github.com/bbci

^4^https://www.uzh.ch/keyinst/loreta.htm
